# Interaction between Styrofoam and Microalgae *Spirulina platensis* in Brackish Water System

**DOI:** 10.3390/toxics9030043

**Published:** 2021-02-26

**Authors:** Hadiyanto Hadiyanto, Amnan Haris, Fuad Muhammad, Norma Afiati, Adian Khoironi

**Affiliations:** 1 Departments of Environmental Science, School of Postgraduate Studies, Diponegoro University, Semarang 50275, Indonesia; amnan.haris43@gmail.com (A.H.); fuad.muh@gmail.com (F.M.); 2 Center of Biomass and Renewable Energy (CBIORE), Chemical Engineering Department, Faculty of Engineering, Diponegoro University, Semarang 50271, Indonesia; 3 Biology Department, Faculty of Sciences and Mathematic, Diponegoro University, Semarang 50271, Indonesia; 4 Fisheries Science, Faculty of Fisheries and Marine Science, Diponegoro University, Semarang 50271, Indonesia; normaafiati.na@gmail.com; 5 Department of Environmental Health, Faculty of Public Health, Dian Nuswantoro University, Semarang 50131, Indonesia; batik.ayacantik@gmail.com

**Keywords:** microplastic pollutant, polystyrene, biodegradation, microalgae

## Abstract

Styrofoam is a thermoplastic with special characteristics; it is an efficient insulator, is extremely lightweight, absorbs trauma, is bacteria resistant, and is an ideal packaging material, compared to other thermoplastics. The aim of this study was to analyze the interaction between Styrofoam and *S. platensis*. The study examined the growth of *S. platensis* under Styrofoam stress, changes in Styrofoam functional groups, and their interactions. The research method was culture carried out in brackish water (12 mg/L salinity) for 30 days. *S. platensis* yields were tested by FTIR and SEM-EDX and Styrofoam samples by FTIR. The results showed the highest growth rate of *S. platensis* in cultures treated with 150 mg Styrofoam that is 0.0401 day^−1^. FTIR analysis shows that there has been a change in the functional group on Styrofoam. At a wavelength of 3400–3200 cm^−1^ corresponds to the alcohol group and there was an open cyclic chain shown by the appearance of a wavelength at 1680–1600 cm^−1^ assignment to alkene. SEM-EDX test results show that Styrofoam can be a resource of nutrition, especially carbon for *S. platensis* to photosynthesize. Increased carbon content of 24.56% occurred in culture, meanwhile, Styrofoam is able to damage *S. platensis* cells.

## 1. Introduction

The increasing human population causes an increase in the amount of plastic waste. Plastic pollution has become a major issue in Sustainable Development Goals (SDGs) and is stated in point number 12, under the header “Responsible Consumption and Production”. Plastics are a material that degrade very slowly and may stay in the environment for a long period [1]. Plastics are available in environment in a wide range of size and forms with different chemical composition, density and color [2,3]. Plastics with microscopic sizes are called microplastics and have a diameter between 1 µm to 5 mm [4,5]. Furthermore, European Chemical Agency (ECHA) [6] defined microplastics as a solid polymer material and their additives or other substances, most of which have particle dimensions of 1 nm to 5 mm, and for fiber form, the size is mostly in the length of 3 nm to 15 mm with length to diameter ratio greater than 3. Auta et al. [7] and Frias and Nash [8] categorized microplastics in aquatic environment into two types: primary microplastics and secondary microplastics. In the first type, they include plastic based products for daily domestic and industrial usages, i.e., personal care products, facial scrubs, insect repellents [3,4,9] as well as products from the ship-breaking industry and air-blasting technology [1,4]. The second type includes smaller fragments of plastic from breaking of larger plastic items in aquatic systems through biological degradation, photo-degradation, chemical deposition, and physical fragmentation [1,3,4,10]. The common microplastics found in aquatic environment are polypropylene, polyethylene, polystyrene, polyvinylchloride, and polyethylene terephthalate [10]. Both types of microplastics present in aquatic environments are reaching certain concentrations and may have effects on aquatic organism including microalgae.

Polystyrene or Styrofoam is a type of plastic with light properties, heat resistance, and low production costs. Until now, Styrofoam is sold freely in shops, stalls, and even supermarkets. Styrofoam is widely used as a food and beverage container. After use, this Styrofoam container would be discarded, though it is still in good condition and can be reused. Most of the consumers lack the knowledge that Styrofoam needs a long time to be completely degraded. Styrofoam can be recycled. However, the high cost and complicated process make producers prefer to produce new Styrofoam, rather than recycle it [11]. Styrofoam is light because 95% of it is air, making it unsinkable [12]. Styrofoam waste is easily caught in dams and aquatic plants. The nontransparent color of Styrofoam can reduce the amount of sunlight entering the water, which makes algal photosynthesis less than optimal [13]. Environmental factors such as weather changes and water micro-organism cause plastic to degrade into microplastics [14]. Microplastics are plastic particles < 5 mm [15] and through the degradation process, the polymer chains in plastics turn into monomers. Frequently, new chemical bonds will also be formed as a byproduct of this process [16]. Microplastics can be found in all parts of the aquatic system [17]. Due to their very small size, microplastics can be ingested by aquatic biota and cause disease [18]. Microplastics also spread through the food chain [19].

Styrofoam consists of long hydrocarbon chains, providing an opportunity for microalgae to use the chemical content in Styrofoam as nutrients. The carbon content in Styrofoam can spur the growth of microalgae. According to Li et al., [14] though polystyrene could inhibit *C. reindhartii* growth, they are still able to adapt because they obtain organic carbon sources from polystyrene and use it for growth. However, there are additive substances in plastics such as Bisphenol-A (BPA), phthalates, trace elements, and refractory substances, which make plastic durable and dangerous, especially for microalgae. One of them is *S. platensis*, which is often used in the food, cosmetic, and medicinal industries. These components of Styrofoam damage *S. platensis* cells, as a result of which photosynthetic activity is decreased and cell growth is inhibited [9].

Microalgae is a photosynthetic microorganism that utilizes carbon source and sunlight for the photosynthesis process lead to biomass production. Microalgae biomass can be extracted for value added products mostly containing protein, lipids, and carbohydrate. Because of their importance, the potential effect of microplastic on their growth must be studied. Microalgae cells of *Chlorella* sp. and *Scenedesmus* sp, are able to absorb nanoplastic beads (0.02 μm) and resulting inhibition of photosynthesis and induction of oxidative stress [20]. Moreover, Khoironi et al. [10] showed that there was an interaction between *Spirulina* sp. cells with microplastics. Microplastic can be absorbed by the *Spirulina* sp. cell and it utilizes them as a source of carbon for photosynthesis. Marquez et al. [21] stated that *S. platensis* is capable of growth on glucose heterotrophically under aerobic-dark conditions and that the photosynthetic activity and oxidative assimilation of glucose can independently operate mixotrophically under light conditions. These phenomena are mainly caused by physical and chemical properties of the microplastics and the morphological and biochemical properties of the algae. Furthermore, Bhattacharya et al. [20] reported that algae and microplastic has a great affinity in which microplastic particles have positive charges.

Microalgae can also produce Extracellular Polymeric Substances (EPS), which stimulates formation of biofilms on the microplastic surface, which is the main indicator of damage to microplastic material. Since biofilms contain nutrients, they can be a suitable living environment for other micro-organisms such as bacteria, fungi, and protozoa. The presence of these micro-organisms will form a protein structure such as enzyme that acts as a metabolic catalyst and breaks down chemical elements in the polymer into other elements. The chemical elements of polymers can form nutrition for micro-organisms, so that the latter obtain two resources of nutrition simultaneously, viz., *S. platensis* biofilms and chemical compounds of microplastic. The ability of micro-organisms to utilize chemical elements from polymers as nutrients is called biodegradation [22], because it will have an impact on changes in the chemical compounds in polymers [11].

Brackish water is found in estuary areas, has its own diversity, and is usually used for aquaculture such as milkfish. Microalgae serving as major producers of aquatic ecosystems are also found here [23]. However, microalgae might also be affected by the presence of microplastics in water bodies [24]. It has been proved that microplastic particles and doses can cause toxic effects on microalgae, including inhibition of growth, decreased photosynthetic efficiency, etc. [10,14,18]. However, the opposite results were also found by some researchers. Sjollema et al. [25] emphasized the impact of microplastic on growth rate, but not on photosynthetic efficiency for marine flagellates *Dunaliella tertiolecta* under a high exposure concentration of 250 mg/L with a particle size of 0.05 mm. Canniff and Hoang [26] showed that plastic microbeads could serve as a substrate for *Raphidocelissubcapitata*, thus, benefiting microalgae growth. Further, high concentrations of microplastics with a size of N400 μm had no deleterious effect on freshwater microalgae *Chlamydomasreinhardtii* [15]. Considering the contradictory discoveries and the limited number of microalgae species tested, more investigation is needed.

This research aims to investigate the inhibitory effects of different dosages of PS microplastics on the growth and photosynthetic efficiency of *S. platensis* and the effect of microalgae on the physical morphology of PS. The results of this study are expected to provide information useful for updating knowledge relating to the toxicity of PS with different dosages in the aquatic environment.

## 2. Materials and Methods

### 2.1. Styrofoam Preparation

The microplastics used in this study were Styrofoam granules obtained from CV. Mitra Sejati Foamindo, Genuk, Semarang City, Indonesia. The Styrofoam was weighed carefully with mass concentrations of 150, 250, and 400 mg in 500 mL culture volume, washed with ethanol and dried at room temperature for 24 h.

### 2.2. Culture Preparation of S. platensis

**Figure 1:**
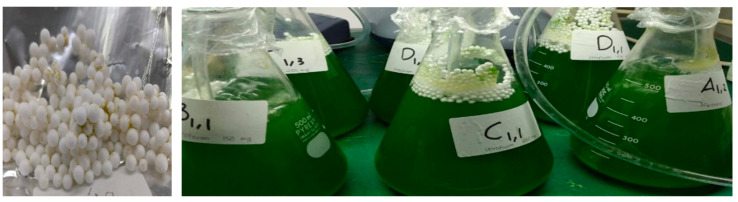
Microplastic Styrofoam with a diameter of 2 mm (**left**) and implementation of Styrofoam in microalgae culture (**right**).

Microalgae *S. platensis* was obtained from Neoalgae, Sukoharjo, Central Java, Indonesia. Microalgae cultivation, testing, and result analysis were carried out at the UPT C-BIORE Laboratory, Diponegoro University, Semarang, Indonesia. Culturing was performed in 500 mL Erlenmeyer glasses, each equipped with an aerator (BS-410, Amara, Shanghai, China) (Figure 1). The cultures were placed into an illumination incubator under an 8W Philips tube lamp with light intensity of 1500 lux (light/dark ratio was 24 h/0 h). The cultivation temperature was controlled at about 23 ± 2 °C. Styrofoam was put into an Erlenmeyer, which already contained the culture of *S. platensis*. The experiment was set up for four different Styrofoam concentrations (*Spirulina* A = *Spirulina* culture without Styrofoam or as a control, *Spirulina* B = *Spirulina* culture with 150 mg Styrofoam, *Spirulina* C = *Spirulina* culture with 250 mg Styrofoam, and *Spirulina* D = *Spirulina* culture with 400 mg Styrofoam). Each culture was conducted in triplicate experiments while the Optical Density (OD) was measured for 30 days. Nutrient was given every two days in the form of a mixture of 15 ppm TSP, 70 ppm Urea, and 1 g/L NaHCO_3_, to maintain the growth of *S. platensis*. OD was measured using a spectrophotometer (OPTIMA SP-300, Osaka, Japan) to determine the density of cells in *S. platensis* under the wavelength of 680 nm. Growth rate (µ) was measured using the formula [27]:

$$µ=\;\frac{lnXn-lnXo}{tn-to}$$

where *ln X* is the natural logarithm of optical density and *t* is the time observed for *S. platensis*.

### 2.3. Harvesting of S. platensis

After a 30-day exposure under the toxicity test (PS microplastics), *S. platensis* was harvested. Before harvesting, Styrofoam was separated by filtering *Spirulina* sp. containing micro plastic with a Whatman filter diameter of 1 mm to obtain *Spirulina* sp. without microplastic. Harvesting of *S. platensis* was carried out on the 30th day of culture by the filtration method. Filtrate obtained was in the form of wet biomass, which was dried in the oven at 35–40 °C temperature. Dry *S. platensis* samples were taken randomly for SEM-EDX analysis and Styrofoam samples for FTIR analysis.

### 2.4. FTIR and SEM Analysis

FTIR is a common technique used to determine any changes in the functional group of Styrofoam and was adopted for investigation of plastic degradation as stated in ISO 4582 and ISO 4892 for UV exposure, and for microorganism’s surface colonization in ISO 846 and ISO 11266 [14]. The Styrofoam plastics that were applied in *Spirulina* sp. were taken every two days for about 30 days. Prior to the FTIR test, plastics were rinsed with distilled water and left to dry for 24 h, then, the Styrofoam was cut at a size of 2 mm. A FTIR apparatus Perkin Elmer Type Frontier (USA) was used to collect spectra from 4000–200 cm^−1^ (SNI 19-4370-2004 method) and ASTM D6288-89. FTIR test was also conducted in *Spirulina* sp, which had interacted with microplastic treatment for 30 days.

The morphology of microplastic Styrofoam was observed using scanning electron microscope (SEM) and combination with Energy Dispersive X-ray spectroscopy (EDX) to determine the inorganic elements contained in the material [14]. The analysis was conducted at room temperature and metalized using Au.A Jeol (model JSM-6510 LA, Tokyo, Japan) at 3000× magnification.

### 2.5. Statistical Analysis

Triplicates were applied and results were presented as means ± standard error of the mean. *S. platensis* growth rate data were statistically analyzed using the IBM SPSS application version 25, using the one-way ANOVA test followed Post-Hoc analysis with a confidence level of 95%. A value of *p* < 0.05 was used to reveal a significant difference.

## 3. Results

### 3.1. Spirulina platensis Growth under Styrofoam Pressure

**Figure 2:**
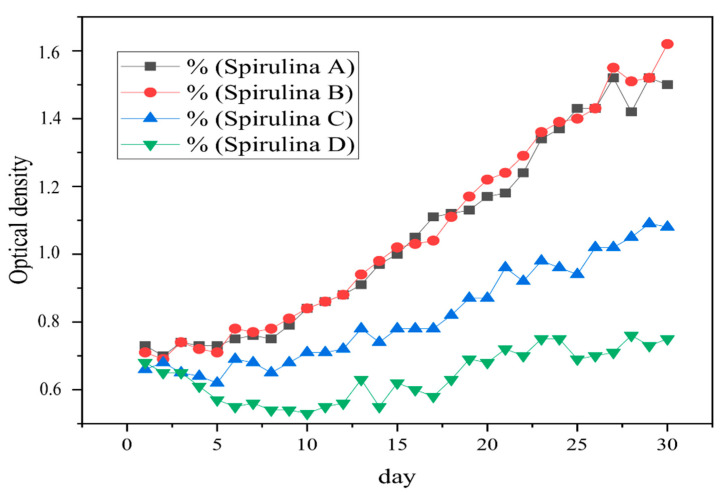
Brackish water culture *S. platensis* growth in each treatment (*Spirulina* A is a control (without Styrofoam), *Spirulina* B = 150 mg Styrofoam/500 mL culture, *Spirulina* C = 250 mg Styrofoam/500 mL culture, *Spirulina* D = 400mg Styrofoam/500 mL culture).

The brackish water cultivation was imbued with 12 mg/L of NaCl, for maintaining the consistency of the culture in a brackish condition until harvest. According to Astuti, Jamali, and Amin [28], brackish water has a salinity of 0.5–17 mg/L. For 30 days, the salinity of the media fluctuates, but still in the brackish water range. *S. platensis* prefers higher salinity conditions. According to Hadiyanto dan Azim [29], *S. platensis* is able to grow in environments of high salinity, because in these conditions, some contaminants such as microbes are not able to survive. The graph of *S. platensis* growth in brackish water culture with Styrofoam treatment can be seen in Figure 2. In Figure 2, there is a point that shows an extreme increase in optical density. Culture A on day 28 from 1.42 to 1.52; culture B on day 29 from 1.52 to 1.62; C culture on day 28 from 1.05 to 1.09 and culture D on day 27 from 0.71 to 0.76. This extreme increase in optical density value shows the *S. platensis* culture experiencing an exponential phase [30].

**Figure 3:**
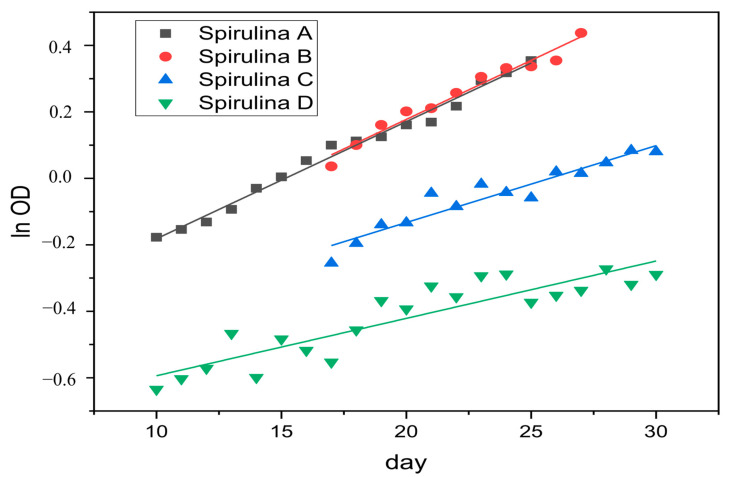
The logarithmic of optical density of *S. platensis* at the exponential phase in brackish water in various concentrations of microplastic treatment (**A**) control, (**B**) 150 mg, (**C**) 250 mg, and (**D**) 400 mg.

In order to evaluate the significance difference between experiments, One-way ANOVA followed by Post-Hoc Tukey HSD (honestly significant difference) was used in this research. Based on Figure 3 and calculation of the means of growth rate constant (µ) of each experiment (Table 1), it was revealed that the growth rate of *S. platensis* A (control) is 0.035925 day^−1^. *S. platensis* B with 150 mg/500 mL Styrofoam treatment was 0.03525 day^−1^. *S. platensis* C treated with Styrofoam 250 mg/500 mL was 0.02675 day^−1^. *S. platensis* D treated with Styrofoam 400 mg/500 mL was 0.020425 day^−1^. Furthermore, Table 2 also shows that the *p*-value (2.295 × 10^−10^) between group corresponding to the F-statistic of one-way ANOVA is lower than 0.05, hence, H0 (null hypothesis 0 is rejected and H1 is accepted [31], indicating a difference in the *S. platensis* growth in brackish water, treated with different levels of Styrofoam.

The Tukey HSD test (Table 3) was then used to identify which pairs of these experiments are significantly different from each other. Comparing experiment A (control) and B (150 mg Styrofoam/500 mL *Spirulina*) revealed that they are insignificantly different of their growth rate as its *p*-value (0.7948595) is higher than 0.01. Moreover, the pairs of experiments A–C, A–D, B–C, B–D, and C–D show significant differences since all the Tukey HSD *p*-value are lower than 0.01 (Table 3).

### 3.2. Styrofoam Degradation

Fourier Transform Infrared (FTIR) is a tool for determining the functional groups and molecular bonds of a chemical compound in a specimen. Its working principle is the interaction between spectrum originating from the source and the test sample material. The sample will generate vibrations, which will be captured by the detector and finally translated into a transmittance curve that has certain peaks with a spectrum of 4000–400 cm^−1^ [32]. In this research, FTIR was employed detect degradation in plastic by considering changes in functional groups [14].

**Figure 4:**
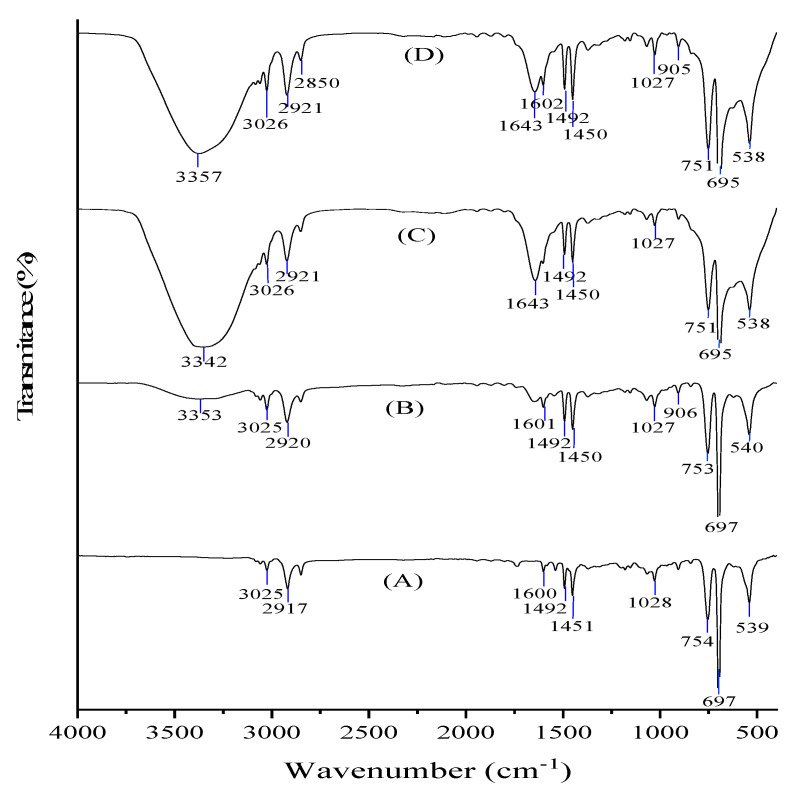
FTIR results of the ratio of Styrofoam (**A**) before treatment, (**B**) 150 mg, (**C**) 250 mg, and (**D**) 400 mg; after 30-day treatment with *S. platensis* in brackish water culture.

Figure 4 shows the effect of presence of microplastics with different concentration in microalgae *Spirulina* sp. culture. According to Dmytryk et al. [33], the wavelength of 3800–3200 cm^−1^ indicates the amine functional group (NH_3_) in the protein. The following peak, 1750–1600 cm^−1^ represents the primary amide and carbonyl (C=O) groups in the protein. The stretching vibrations observed in the frequency range of peaks 1450 cm^−1^ and peaks at 1400–1300 cm^−1^ represent carboxyl (COO-) and alkyl groups, respectively. Then at a wavelength of 1050–1000 cm^−1^ stretching of CO, CC, and OH in the presence of ether, ester, and hydroxyl of polysaccharides are observed.

Furthermore, Figure 4 shows that no O-H groups in Styrofoam, which was found also in brackish water Styrofoam, where peaks (3353 cm^−1^) began to form with low intensity. O-H groups were clearly visible in Styrofoam C, D-brackish water. The peak read was in the range of 3378–3345 cm^−1^ with an intensity of 59.18–67.65%. The presence of an O-H group also has been confirmed with a C-O group (1300−1000 cm^−1^). Which can be seen in brackish water B, C, D-Styrofoam. This shows a change in the functional group on Styrofoam, with evidence of the formation of an alcohol group (-COOH) [11].

### 3.3. Interaction of S. platensis with Styrofoam

Scanning Electron Microscopy (SEM) is a tool for determining the surface morphology of a specimen, including changes caused by micro-organisms [34]. SEM performance using a magnification of 3000× is supported by EDX, which is able to determine the content of inorganic elements in a specimen using X rays [35].

**Figure 5:**
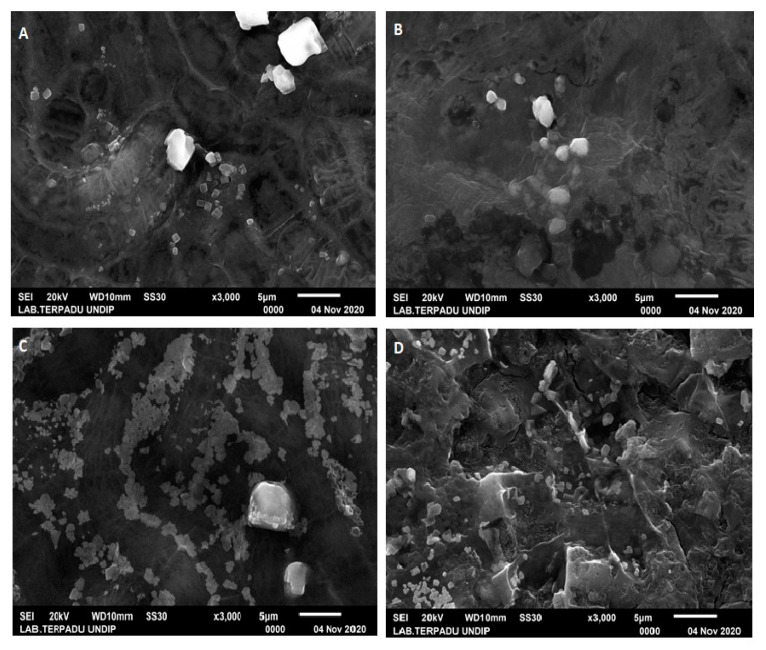
SEM analysis results of brackish water culture *S. platensis* for 30 days. (**A**) *S. platensis* without Styrofoam treatment. (**B**) *S. platensis* treated with Styrofoam 150 mg/500 mL. (**C**) *S. platensis* treated with Styrofoam 250 mg/500 mL. (**D**) *S. platensis* treated with Styrofoam 400 mg/500 mL.

SEM analysis results on brackish water *S. platensis* showed that around the *S. platensis* A, B, C, and D, cells produced EPS in the form of small spheres and large nuggets, thought to be salt or urea given during culture (Figure 5). Further, the morphology of *S. platensis* A was still normal, while *S. platensis* B, C, and D were seen to be damaged. According to Li et al., [4] the presence of microplastics can damage microalgae cell membranes, thus inhibiting the photosynthesis process.

The results of EDX analysis (Table 4) on brackish water *S. platensis* showed that in culture B and C, there was an increase in carbon content, namely 24.56% and 4.24%, compared to *S. platensis* A culture, whereas in D culture, there was a decrease in carbon content by 2.14%.

## 4. Discussions

Our research reported an interaction between microalgae and Styrofoam microplastic. Infusion of Styrofoam had an impact on the S. platensis growth rate, because Styrofoam gave a shading effect on the culture surface, thereby reducing the light intensity used by *S. platensis* for photosynthesis [15]. Imposing Styrofoam 150 mg in 500 mL *Spirulina* culture did not significantly affect the growth rate as compared to control (Figure 2), which means that at this concentration the Styrofoam did not give a shading effect and eventually microalgae cell could use carbon from the Styrofoam (Table 1 and Table 2). However, increasing Styrofoam concentration (250 mg/500 mL and 400 mg/500 mL) the growth of algae cell was significantly retarded by the Styrofoam particles concentration (Figure 2). Moreover, the decrease in the growth rate of S. platensis may be also influenced by the formation of excess Extracellular Polymeric Substances (EPS), which is toxic to *S. platensis* itself. The presence of EPS will be a place for other micro-organisms to compete with algae cells in the absorption of nutrients, both from the culture and from the breakdown of carbon chains from Styrofoam [14].

The growth rate of *S. platensis* B culture (given Styrofoam 150 mg/500 mL) in brackish waters was the highest as compared to 250 mg/500 mL and 400 mg/500 mL. This is presumably because *S. platensis* obtains additional nutrients from the degradation of Styrofoam (Table 1 and Figure 3). In addition, the Styrofoam in culture B did not cover the entire surface of the culture, so that the light could still enter and be used properly by *S. platensis*. Increased levels of Styrofoam resulted in a decrease in the growth rate of the *S. platensis* culture as evidenced by culture D, which has a lower growth rate than culture C, due to *S. platensis* being under pressure from the environment in the form of Styrofoam. The number of Styrofoam floating on the surface is also able to block light from entering the culture, thus, inhibiting the photosynthesis process [15].

The FTIR analysis (Figure 4) depicts that no carboxyl groups (C=O, at a wavelength of 1810−1630 cm^−1^) are formed, indicating the absence of oxidation reaction to Styrofoam. The structure of Styrofoam showed the presence of an aromatic C=C group and no aliphatic C=C group was formed, indicating that the initial structure of Styrofoam in the form of styrene has a closed chain (cyclic) shape. However, all the FTIR test results on Styrofoam that were included in the brackish water *S. platensis* culture, showed the presence of aromatic C=C groups and aliphatic C=C groups, proving that there is an open cyclic chain [11]. Mohamed et al. [32] stated that Styrofoam is stable because its constituent structure is a cyclic chain with a very long arrangement. The opening of the cyclic chain proves the occurrence of degradation, although such degradation has not yet reached physical fragmentation and changes into simpler chemical monomers [14]. Another phenomena showed that all FTIR in Styrofoam showed a peak at a wavelength of 754–538 cm^−1^ with a sharp peak at 697–695 cm^−1^. According to Nandiyanto, Oktiani, and Ragadhita [31], the peak of 750 cm^−1^ is a characteristic of aromatic compounds. These FTIR test data results on concluded that Styrofoam has interaction with *S. platensis* cells in the culture. According to Chentir et al. [36], increasing the concentration of NaCl can reduce the availability of nutrients such as nitrogen, thereby triggering the incorporation of carbon both from *S. platensis* and from Styrofoam into EPS. The decrease in carbon content in algae culture indicates damage to the cell membrane of *S. platensis*, which affects the ability of photosynthesis. Li et al. [4] stated that although microalgae are able to absorb carbon from plastics, these plastics are at risk of damaging cell membranes; hence, plastic is not a good source of nutrition for microalgae.

Styrofoam is composed of styrene chains, which are a source of carbon for micro-organisms in the waters. This causes the nutrients needed for photosynthesis of *S. platensis* especially from the element carbon supplied by Styrofoam, which is available in the medium. The availability of this carbon can support the growth of *S. platensis*, which will have an impact on increasing the production of Extracellular Polymeric Substances (EPS), which in turn plays a role in producing a biofilm on the Styrofoam surface [37,38]. Biofilms are a suitable abode for other micro-organisms such as bacteria, fungi, protozoa etc., which play a role in the degradation of the Styrofoam surface. During this microbial activity, micro-organisms will form protein structures in the form of enzymes that play a role in changing the chemical content in Styrofoam into other forms. The presence of other inorganic elements in the EDX analysis proved that *S. platensis* was able to absorb contaminants, which can come from the release of additives from Styrofoam, such as Mg, Al, Si, S, Ca, K, Cl, Cr, Zn, Cu etc., as well as from the nutrients given such as C, N, P, Na, Cl etc. [14].

## 5. Conclusions

This interaction between Styrofoam and microalgae *Spirulina* sp. has been investigated in this research. The growth of microalgae, the change of morphological structure of Styrofoam and chemical functional groups were measured and used in determining the effect of interactions. The results of the variations of Styrofoam concentration from 300 g/L to 800 g/L in microalgae culture showed significant inhibitory effects on *Spirulina* sp. growth. There was a change in the functional group on Styrofoam as an indicator of biodegradation, with evidence of the formation of an alcohol group (-COOH) at a wavelength of 3400–3200 cm^−1^ and an open cyclic chain (peaks appearing at a wavelength of 1680–1600 cm^−1^). SEM-EDX test results show that Styrofoam can be a source of nutrients, especially carbon, needed by *S. platensis* for photosynthesis. However, the presence of microplastic Styrofoam also gives a deterioration effect to the microalgae cell, which cause photosynthetic inhibition. The findings of this work essentially improve understanding of the interaction between microplastics and microalgae cell in aquatic environments. The continuous influence of different sizes of microplastics on microalgae or other organisms should be further investigated. Nevertheless, this study only showed the preliminary findings on the interaction between Styrofoam with microalgae and further investigation and detail analysis should be done in more replications experiments to obtain a statistical significance of the results.

## Figures and Tables

**Table 1:** Means value and their variances of each experiment.

Treatments	Sum	Average µ (day^−1^)	Variance
Control (A)	0.1437	0.035925	1.2425 × 10^−6^
150 mg/500 mL (B)	0.141	0.03525	0.00000259
250 mg/500 mL (C)	0.107	0.02675	0.00000055
400 mg/500 mL (D)	0.0817	0.020425	2.49167 × 10^−7^

**Table 2:** Analysis of variance (ANOVA) of F and *p* values between experiments.

Source of Variation	df	MS	F	*p*-Value	F Crit
Between Groups	3	0.000218974	189.1104714	2.2956 × 10^−10^	3.49029482
Within Groups	12	1.15792 × 10^−6^			
Total	15				

df, degree of freedom; MS, Mean Square is just the Sum of Squares divided by its degrees of freedom, and the F value is the ratio of the mean squares.

**Table 3:** The post-hoc Tukey HSD analysis of four group experiment.

Treatment Pair	Tukey HSD Q Statistic	Tukey HSD *p*-Value	Tukey HSD Interfence
A–B	1.2546	0.7948595	insignificant
A–C	17.0529	0.0010053	** *p* < 0.01
A–D	28.8087	0.0010053	** *p* < 0.01
B–C	15.7983	0.0010053	** *p* < 0.01
B–D	27.5541	0.0010053	** *p* < 0.01
C–D	11.7558	0.0010053	** *p* < 0.01

**, significant.

**Table 4:** Energy Dispersive X-ray spectroscopy (EDX) analysis results for the chemical constituents of *S. platensis* cultured in brackish water for 30 days.

*S. platensis* Content	Styrofoam Levels			
	*S. platensis* A (Control)	*S. platensis* B + 150 mg	*S. platensis* C + 250 mg	*S. platensis* D + 400 mg
Carbon, C	64.3	85.23	67.15	62.92
Nitrogen, N	18.59	-	16.5	23.69
Natrium Oxide, Na_2_O	4.14	5.39	5.14	3.75
Magnesium Oxide, MgO	0.51	0.2	0.27	0.43
Alumina, Al_2_O_3_	-	-	-	-
Silica Dioxide, SiO_2_	-	-	-	0.31
Phosphor Pentoxide, P_2_O_5_	2.29	1.67	1.75	2.55
Sulfide, SO_3_	1.95	2.15	2.3	1.76
Chloride, Cl	4.56	3.58	4.9	2.8
Kalium Oxide, K_2_O	3.67	1.78	1.99	1.78
Calcium Oxide CaO	-	-	-	-
Cuprum (II) Oxide, CuO	-	-	-	-
Zinc Oxide, ZnO	-	-	-	-

## Data Availability

The data presented in this study are available on request from the corresponding author.
